# Changes in Physicochemical Properties and Antioxidant Activities of Persimmon Wine During Fermentation

**DOI:** 10.3390/foods14162763

**Published:** 2025-08-08

**Authors:** So-Won Jang, Hwan Hee Yu, Da-Sol Jung, Jong-Chan Kim, Jae Hoon Lee, Mi Jang

**Affiliations:** 1 Food Standard Research Center, Korea Food Research Institute, 245 Nongsaengmyeong-ro, Wanju-gun 55365, Republic of Korea; wish@kfri.re.kr (S.-W.J.); yhh@kfri.re.kr (H.H.Y.); dsjung@kfri.re.kr (D.-S.J.); jckim@kfri.re.kr (J.-C.K.); 2 Department of Food Science & Technology, Jeonbuk National University, 567 Baekje-daero, Deokjin-gu, Jeonju-si 54896, Republic of Korea; joynjh@jbnu.ac.kr

**Keywords:** persimmon wine, alcoholic fermentation, physicochemical properties, methanol, antioxidant

## Abstract

In this study, persimmons, which are rich in various nutrients and bioactive compounds, were used as the raw material for wine production. Persimmon wine was produced by inoculating with *Saccharomyces cerevisiae* and fermenting the mixture at 30 °C for seven days. During this process, we analyzed changes in physicochemical properties, organic acids, free sugars, ethanol, methanol, free amino acids, total phenolic content (TPC), total flavonoid content (TFC), and antioxidant activities. Over the seven-day fermentation period, soluble solids decreased from 16.27 °Brix to 5.57 °Brix, pH declined from 5.93 to 4.90, and total acidity increased from 0.12% to 0.41%. Succinic, lactic, and acetic acids were identified as major organic acids, while glucose and fructose were depleted after six days of fermentation. The ethanol concentration reached 7.93% on day seven, while methanol increased to 0.050%. The free amino acid content decreased as fermentation progressed. The TPC and TFC increased significantly. Antioxidant capacity increased, as observed from DPPH, ABTS, and FRAP assay results. These results suggest that alcohol fermentation of raw persimmons induces significant changes in key quality-related components and enhances antioxidant activity. Overall, the findings provide valuable foundational data supporting the industrial application of persimmon wine.

## 1. Introduction

Persimmons (*Diospyros kaki* Thunb.) are rich in dietary fiber and are predominantly cultivated in several Asian countries, notably China, Korea, and Japan [1]. More than 5.75 million tons are produced globally each year, with approximately 85% being produced in Asian countries [2]. Persimmons are rich in vitamins A, B, and C and polyphenols, including gallic acid, catechin, and quercetin, which possess potent antioxidant, anti-inflammatory, and anticancer activities [3,4]. In addition, persimmons are rich in glucose and fructose and contain substantial amounts of amino acids, such as proline and glutamic acid, making them highly suitable as fermentation substrates [5,6]. These fruits are consumed as fresh produce and in processed forms, such as dried persimmons, semi-dried persimmons, and persimmon vinegar [7,8]. Recent studies have focused on increasing their added value through the process of fermentation.

Fermentation is a biochemical process in which microorganisms utilize carbohydrates to produce various metabolites, such as alcohols, organic acids, and carbon dioxide. Through this process, the taste, aroma, texture, and shelf life of raw materials are enhanced [9]. Among various types of fermentation, alcoholic fermentation by yeast involves the breakdown of monosaccharides, such as glucose and fructose, into ethanol and carbon dioxide. As fermentation progresses, changes in organic acid composition influence the acidity and flavor profile, while the rate of sugar consumption significantly affects the fermentative efficiency of yeast and the final ethanol concentration [10,11,12]. The high sugar content of persimmons promotes ethanol production; however, during fermentation, pectin components in persimmons may be degraded by yeast or enzymatic activity, leading to the formation of methanol, a potentially harmful compound [13]. Methanol is a toxic, colorless, and volatile liquid that is converted into formaldehyde and formic acid in the human body. These metabolites inhibit mitochondrial cytochrome oxidase activity, potentially causing neurological damage and various metabolic disorders [14]. Compared with ethanol, methanol has a lower affinity for alcohol dehydrogenase, resulting in slower metabolism. When ethanol is present, methanol metabolism is further suppressed, increasing the risk of methanol accumulation in the body [15,16]. Therefore, in fermented wines with high ethanol contents, it is essential to monitor and control methanol levels to ensure safety. In addition, antioxidant activity can increase during fermentation due to the generation or release of bioactive compounds, serving as an important indicator to evaluate the functional quality of wine. Although methanol formation and antioxidant activity are key factors representing safety and functionality, respectively, studies that simultaneously analyze these parameters to comprehensively assess the quality and safety of persimmon wine are scarce. Previous studies on persimmon-based wine production have primarily focused on physicochemical properties or changes in specific bioactive compounds, with limited research addressing both methanol formation and antioxidant activity during fermentation.

This study aimed to produce persimmon wine by inoculating *Saccharomyces cerevisiae* into persimmon mash and analyzing changes in physicochemical characteristics, organic acids, free sugars, ethanol, methanol, free amino acids, antioxidant compounds, and antioxidant activity throughout the fermentation process. The results of this study offer essential information that may contribute to enhancing the quality and guaranteeing the safety of persimmon wine.

## 2. Materials and Methods

### 2.1. Production of Persimmon Wine

Cheongdo-Bansi (*Diospyros kaki* Thunb. cv. Cheongdo-Bansi) is a representative cultivar of astringent persimmon produced in Cheongdo, Gyeongsangbuk-do, Republic of Korea. The fruit is seedless and has soft flesh and a high sugar content. In this study, Cheongdo-Bansi persimmons harvested in November 2024 were obtained and fermented in the laboratory. The fruits were washed under running water to eliminate surface impurities and fungal mycelia. The calyx and any deteriorated parts were removed before homogenization. A total of 25 kg of pretreated persimmon mash was transferred to a fermenter, and 10 g of yeast was inoculated. For alcoholic fermentation, *S. cerevisiae* Fermivin, a widely used strain in wine production, was employed. The yeast was activated in sterile distilled water (30 °C, 200 rpm, 30 min). Fermentation was performed at 25 °C for seven days. Persimmon wine was collected at 24 h intervals and analyzed for physicochemical properties using high-performance liquid chromatography (HPLC; organic acids, free sugars, ethanol, methanol, and free amino acids). For the analysis of antioxidant content (TPC, TFC) and antioxidant activities (DPPH, ABTS, and FRAP assay), the fermented samples were concentrated under reduced pressure, freeze-dried into powder, and then dissolved in distilled water for use in assays. Since this study investigated changes during the fermentation period of persimmon wine using *S. cerevisiae*, persimmon juice (day 0) was set as the control.

### 2.2. Physicochemical, Microbiological, and Physical Properties

The °Brix of the fermented samples was determined using a digital refractometer (PR-101α, Atago, Tokyo, Japan), and the pH was analyzed using a pH meter (Orion Star A214; Thermo Fisher Scientific, Waltham, MA, USA). Total acidity (TA) was determined by titrating 20 mL of a 10-fold diluted sample with 0.1 M NaOH, using phenolphthalein. The titration endpoint was indicated by the persistence of a faint pink color. Total acidity was calculated using the following formula:

TA (%) = (V × F × 0.006 × D)/S × 100

where V is the NaOH volume (mL), F is the 0.1 M NaOH factor, D is the dilution factor, and S the sample volume (mL).

Yeast cell counts were determined by 10-fold serial dilution with sterile saline, followed by spreading on potato dextrose agar (PDA; Difco Laboratories, Detroit, MI, USA). After incubation at 30 °C for 48 h, the colony counts were expressed as log CFU/mL.

Solids were measured using the direct drying method at 105 °C. Specific gravity was measured using a hydrometer, and 10 μL of each sample was used for the measurement.

### 2.3. Organic Acid, Free Sugar, and Ethanol Contents

HPLC analysis (Jasco, Tokyo, Japan) was carried out according to the method described by Klein and Leubolt [17]. Organic acids were analyzed using an Aminex HPX-87H column with10 mM sulfuric acid (H_2_SO_4_) as the mobile phase (0.6 mL/min). The column was maintained at 55 °C, and detection was carried out at 210 nm with a UV detector (UV-975, Jasco, Tokyo, Japan). Free sugars and ethanol were analyzed using the same column with 5 mM sulfuric acid (0.6 mL/min) at 60 °C, and detection was performed using a refractive index (RI) detector (RI-1530, Jasco, Tokyo, Japan). Calibration curves were constructed using standard solutions of organic acids (oxalic acid, citric acid, succinic acid, lactic acid, and acetic acid), free sugars (glucose and fructose), and ethanol.

### 2.4. Methanol Contents

Methanol content was determined by GC-FID (GC-2010, Shimadzu, Kyoto, Japan) according to the method described by Kim et al. [18]. For calibration, external standard solutions of methanol were prepared in various concentrations using dimethyl sulfoxide (DMSO) as the solvent. Hexanol was used as the internal standard. The same concentration of hexanol was also added to the external standard solutions for quantitative analysis.

For sample preparation, 1 g of sample was subjected to extraction with 1 mL hexanol (internal standard) and DMSO to a final volume of 10 mL, shaken (1300 rpm, 60 min), centrifuged (1300 rpm, 15 min), and filtered (0.45 μm nylon syringe filter). The supernatant was analyzed using an Agilent DB-WAX column (60 m × 320 μm I.D., 0.25 μm film thickness, USA), with helium (He) as the carrier gas.

### 2.5. Free Amino Acid Contents

Free amino acids were analyzed by HPLC-FLD (LC-4000, JASCO, Tokyo, Japan) using a modified method from Dai et al. [19]. The standard solution consisted of 17 amino acids (arginine, histidine, isoleucine, leucine, phenylalanine, proline, and threonine), using a commercially available amino acid standard mixture (WAT088122, Waters Corporation, Milford, MA, USA).

The following reagents were used for analysis: Internal Standard Stock Solution (ISSS, 2.5 mM α-aminobutyric acid, 6.45 mg/25 mL in 0.1 M HCl), Internal Standard Solution (ISS, 20 μL ISSS mixed with 980 μL of 20 mM HCl), and calibration standard/IS mixture (CS/IS, composed of 40 μL ISSS, 40 μL calibration standard, and 920 μL HPLC-grade distilled water). For the standard preparation, 40 μL of the amino acid standard was mixed with 960 μL of HPLC-grade water.

Amino acid derivatization was performed using the AccQ-Fluor reagent kit (WAT052880, Waters Corporation, Milford, MA, USA). The heating block was preheated to 55 °C, and one vial of AccQ-Fluor Reagent Powder (2A) was dissolved in 1 mL of AccQ-Fluor Reagent Diluent (2B) by vortexing for 10 s and heating at 55 °C for up to 10 min until the powder was completely dissolved. For derivatization, 80 μL of AccQ-Fluor Borate Buffer was mixed with 20 μL of the prepared AccQ-Fluor Reagent.

For derivatization of the standard solution, 10 μL of the CS/IS mixture was combined with 70 μL of AccQ-Fluor Borate Buffer and 20 μL of AccQ-Fluor Reagent, reacted at room temperature for 1 min and then heated at 55 °C for 10 min. Fermented wine samples were derivatized using the same procedure: 10 μL of the sample was combined with 20 μL of ISS, 60 μL of AccQ-Fluor Borate Buffer, and 20 μL of AccQ-Fluor Reagent.

The HPLC conditions for the analysis of free amino acids in fermented wine were as follows: Separation was performed using a Waters AccQ-Tag column (3.9 mm × 150 mm, 4 μm, WAT052885, Waters Corporation, Milford, MA, USA). The mobile phases were composed of solvent A (0.1 L acetate–phosphate buffer mixed with 900 mL distilled water) and solvent B (acetonitrile), applied under a gradient elution program. The gradient conditions were as follows: A:B = 100:0 (%) at 0 min, 99:1 (0.5 min), 95:5 (18 min), 91:9 (19 min), 86:14 (27 min), 65:35 (43 min), returning to 100:0 (46 min), and maintained until 50 min.

Analysis was performed with a 10 μL injection volume at a flow rate of 1.0 mL/min, with a 37 °C column temperature, 45 min run time, and FLD detection at 250 nm excitation and 395 nm emission.

### 2.6. TPC

TPC was measured as described by Zhang et al. [20]. A 20 μL sample was reacted with 100 μL of 10% Folin–Ciocalteu’s reagent for 5 min, followed by 80 μL of 7.5% sodium carbonate (Na_2_CO_3_). After 1 h in the dark, absorbance was read at 765 nm. The calibration curve (y = 0.0046x + 0.0184, R^2^ = 0.9994) was generated using gallic acid as the standard and presented as mg gallic acid equivalents (GAE)/g of sample.

### 2.7. TFC

TFC was measured as described by Ochieng et al. [21]. A 100 μL sample was reacted with 10 μL of 5% sodium nitrite (NaNO_2_) for 5 min. Subsequently, 10 μL of 10% aluminum chloride (AlCl_3_) and 80 μL of 1 M NaOH were added and read at 510 nm. The calibration curve (y = 0.0018x − 0.0156, R^2^ = 0.9995) was constructed using catechin as the reference standard. TFC was calculated and presented as mg catechin equivalents (CE)/g of sample.

### 2.8. Antioxidant Activity

DPPH radical scavenging activity was measured using a method from Magalhães et al., with some modifications [22]. Briefly, 50 μL of the sample and 200 μL of 150 μM DPPH solution were added to a 96-well plate and incubated in the dark for 20 min. Absorbance was measured at 517 nm, and scavenging activity was calculated as the percentage decrease in absorbance compared to the control (without sample). The concentration required to achieve 50% scavenging activity (IC_50_) was reported as mg/g of sample.

ABTS radical scavenging activity was determined as described by Le Grandois et al. [23]. ABTS radicals were generated by mixing 7 mM ABTS with 2.45 mM potassium persulfate and incubating in the dark for 16 h. The solution was then diluted with ethanol to an absorbance of 0.70 ± 0.05 at 414 nm. For the assay, 10 μL of the sample was added to 200 μL of the ABTS solution and reacted in the dark for 15 min. Absorbance was measured at 414 nm, and scavenging activity was calculated as the percentage decrease in absorbance compared to the control (without sample). The concentration required to achieve 50% scavenging activity (IC_50_) was reported as mg/g of sample.

The FRAP assay was performed according to Benzie and Strain [24]. The FRAP reagent was prepared by mixing 300 mM acetate buffer (pH 3.6), 10 mM TPTZ (in 40 mM HCl), and 20 mM FeCl_3_ (10:1:1, *v*/*v*/*v*). For the assay, 25 μL of the sample was added to 175 μL of the FRAP reagent in a 96-well plate and incubated at 37 °C for 30 min. Absorbance was read at 595 nm. A calibration curve (y = 0.0055x − 0.0003, R^2^ = 1.0000) was constructed using FeSO_4_·7H_2_O. The results were reported as g FeSO_4_ equivalents/g of sample

### 2.9. Statistical Analysis

Experiments were conducted in triplicate, and the results were presented as means ± standard deviation (SD). Statistical analyses were performed using the SPSS package program (Version 12.0K, SPSS Inc., Chicago, IL, USA). All datasets were first tested for normality using the Kolmogorov–Smirnov test and for homogeneity of variances using Levene’s test, before applying one-way ANOVA to assess differences among samples. Duncan’s multiple range test was used to identify significant variations at *p* < 0.05.

## 3. Results and Discussion

### 3.1. Changes in Physicochemical Properties of Persimmon Wine During Fermentation

During alcoholic fermentation of persimmons with the calyx removed, changes in °Brix, pH, total acidity, yeast cell count, solids content, and specific gravity were analyzed over the fermentation period (Table 1). The °Brix of persimmon wine was 16.27 ± 0.06 °Brix on day 0, which decreased sharply to 8.80 ± 0.00 °Brix on day 2 and further declined to 5.57 ± 0.06°Brix by day 7. The Brix value of persimmon wine decreased significantly as alcoholic fermentation progressed (*p* < 0.05).

The pH and total acidity of persimmon wine changed from 5.93 ± 0.01 and 0.12 ± 0.01%, respectively, on day 0 to 4.90 ± 0.01 and 0.41 ± 0.03%, respectively, on day 7 of fermentation. The pH decreased significantly until day 3 (*p* < 0.05), after which the changes were minimal and not statistically significant. In the case of total acidity, a significant increase was observed from day 0 to day 2 (*p* < 0.05), but the changes from day 3 onward were minor and not statistically significant. According to a study by Kwon et al. [25], the initial pH of persimmon wine ranged from 5.0 to 6.0 and decreased to around 4.2 by the end of fermentation, while the total acidity increased to 0.5–0.6%, which agrees with the findings of this study.

The yeast cell count in persimmon wine increased from 6.63 ± 0.22 log CFU/mL (0 day) to 7.12 ± 0.03 log CFU/mL (day 7). However, toward the end of fermentation, yeast activity declined due to the depletion of fermentable sugars, resulting in no further growth. This is likely due to the limitation of yeast growth caused by the reduction in sugars, which serve as a primary carbon and energy source during fermentation [26].

The solid content of persimmon wine decreased significantly from 16.68 ± 0.08% on day 0 to 2.05 ± 0.09% on day 7 (*p* < 0.05). This decrease is explained by the yeast’s use of carbohydrate and nitrogen compounds as fermentation substrates [27]. Additionally, the decrease in solid contents may have resulted from the conversion of water-soluble components within the persimmon tissue into the liquid phase during fermentation or from the enzymatic degradation of cell wall constituents.

The specific gravity of the persimmon wine decreased from 1.118 ± 0.019 on day 0 to 1.005 ± 0.029 on day 7, which was likely due to the production of ethanol, which has a lower density than water [28]. In general, alcoholic fermentation begins with an initial specific gravity above 1.050, and as fermentation progresses, the value approaches 1.000. A stable specific gravity is commonly used as an indicator of fermentation completion [29,30]. Specific gravity serves as an indirect quality indicator of fermentation progress, as it decreases when yeast consumes sugars and produces ethanol [31]. Therefore, the observed reduction in specific gravity indirectly confirms that alcoholic fermentation by yeast proceeded appropriately in persimmon wine.

### 3.2. Changes in Organic Acids, Free Sugars, Ethanol, and Methanol Contents

The results for organic acids, free sugars, ethanol, and methanol contents in persimmon wine during fermentation are shown in Table 2. The detected organic acids included oxalic acid, citric acid, succinic acid, lactic acid, fumaric acid, and acetic acid, with succinic acid, lactic acid, and acetic acid being identified as the predominant components. In particular, lactic acid increased markedly from 0.00 mg/mL on day 0 to 2.75 ± 0.01 mg/mL on day 7. This increase is presumed to result from lactic acid fermentation by lactic acid bacteria that are naturally present on the surface of persimmons [32]. This trend is consistent with the findings of Lee and Kim [33], who reported that citric acid, lactic acid, and acetic acid levels continuously increased during alcoholic fermentation of persimmons.

Regarding free sugars, the concentrations of glucose and fructose on day 0 were 66.06 ± 0.47 mg/mL and 84.27 ± 0.36 mg/mL, respectively. As fermentation progressed, glucose was completely consumed by day 4, and fructose was fully depleted by day 6 (Table 2). This pattern is consistent with previous findings indicating that yeast preferentially utilizes glucose before fructose during fermentation [34].

The ethanol content of persimmon wine was 0.00% on day 0 and increased significantly to 7.93 ± 0.00% by day 7 (Table 2). Wei et al. [13] reported that persimmon wines that had been fermented with different yeast strains reached ethanol concentrations ranging from 5.8% to 7.4%. Similarly, Wang et al. [2] observed ethanol levels of approximately 5% in persimmon wine. The ethanol content recorded in this study is consistent with previous findings. Alcoholic fermentation by yeast converts one molecule of glucose into two molecules of ethanol and carbon dioxide via the Embden–Meyerhof–Parnas (EMP) pathway, with approximately 51.1% of the fermentable sugar being converted into ethanol [35]. In this study, the initial °Brix was 16.27 ± 0.06, which corresponds to a theoretical ethanol yield of approximately 7–8%. The measured ethanol content of 7.93% closely matched this theoretical prediction.

Methanol is produced during fermentation through the degradation of pectin, which is present in fruits, by yeast or enzymatic activity. Although it shares similar physical properties with ethanol, methanol is converted in the human body into formaldehyde and formic acid, both of which are toxic [36]. Therefore, regulatory agencies in various countries have established safety limits for methanol content. In Korea, the legal limit for methanol in fruit wine is 1 mg/mL, while the International organization of vine and wine(OIV) limits methanol contents up to 400 μg/mL in wine [37,38]. In this study, the methanol content in persimmon wine increased from 0.046 ± 0.00% on day 0 to 0.050 ± 0.00% (Table 2), remaining well below the regulatory limits established both domestically and internationally. Lee et al. [39] reported that the methanol content in persimmon wine ranged from 935 to 949 μg/mL, depending on the yeast strain used. Similarly, Wei et al. [13] reported methanol levels ranging from 81.63 to 401.5 mg/L in persimmon wine produced using different yeast strains, suggesting that methanol production varies depending on the yeast type and fermentation conditions. Therefore, the persimmon wine produced in this study demonstrated an increase in organic acids and ethanol content during fermentation while maintaining methanol levels that were below safety thresholds, indicating acceptable quality and safety.

### 3.3. Changes in Free Amino Acid Contents

The changes in free amino acid contents in persimmon wine during fermentation are shown in Table 3. Detected amino acids included histidine, arginine, threonine, proline, isoleucine, leucine, and phenylalanine, all of which gradually decreased as fermentation progressed. Among them, proline was the most abundant on day 0, with a concentration of 254.76 ± 92.39 μg/mL, which decreased significantly to 62.97 ± 46.85 μg/mL by day 7. Histidine, arginine, threonine, isoleucine, and phenylalanine were completely depleted by day 2, while leucine was fully consumed by day 1 (Table 3). This overall decline in free amino acids was likely associated with the metabolic characteristics of yeast, which preferentially utilize amino acids as nitrogen sources during the early stages of fermentation [40,41]. Thus, the decline in amino acid levels noted in this study was likely associated with yeast nitrogen metabolism and may act as an indirect marker of fermentation progress and yeast activity.

### 3.4. Antioxidant Activity

**Figure 1:**
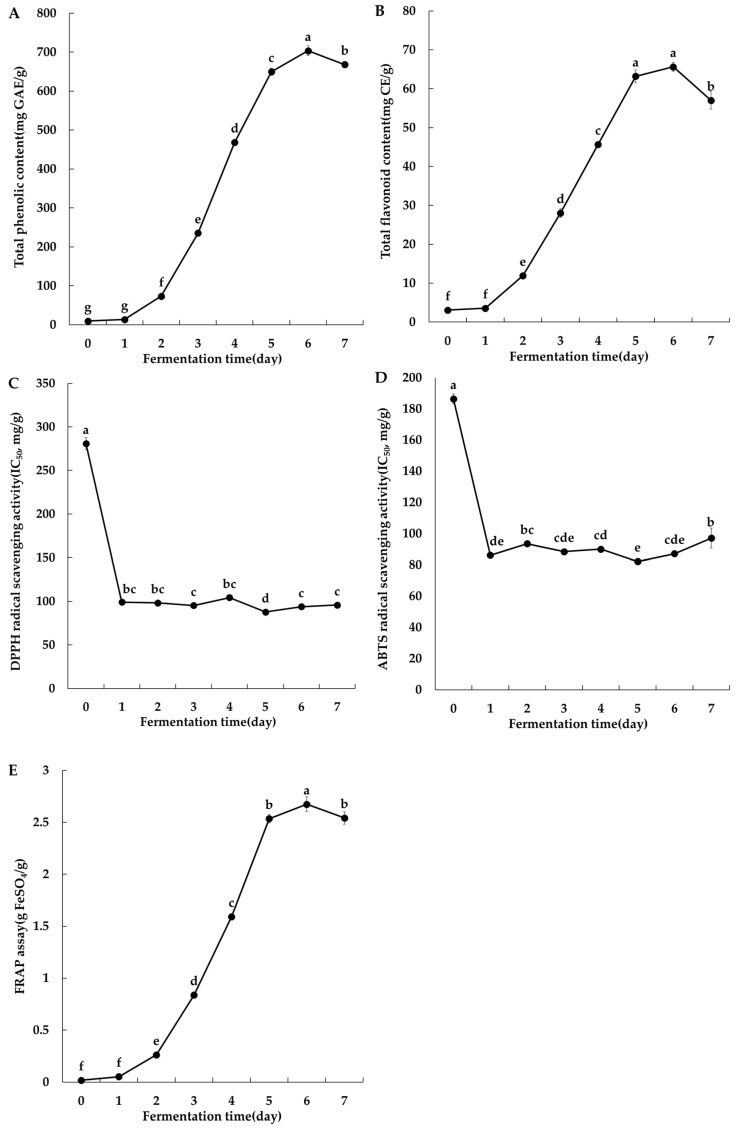
Changes in antioxidant activity in persimmon wine during fermentation. (**A**) Total phenolic content, (**B**) total flavonoid content, (**C**) DPPH radical scavenging activity, (**D**) ABTS radical scavenging activity, and (**E**) FRAP assay results. IC_50_ is defined as the necessary sample concentration for scavenging 50% of free radicals; lower values indicate higher antioxidant activity. Results are expressed as means ± SD (n = 3). Error bar indicates the standard deviation of the mean. Different letters indicate significant differences according to Duncan’s test (*p* < 0.05).

The variations in antioxidant compounds and antioxidant activity of persimmon wine throughout the fermentation process are presented in Figure 1. The freeze-drying yield varied depending on the fermentation day, ranging from 2.7% to 16.33%. Accordingly, all functional measurements using freeze-dried persimmon were expressed on a dry weight basis, calculated by applying the specific yield corresponding to each fermentation day. The TPC and TFC on day 0 were 9.34 ± 0.19 mg GAE/g and 3.06 ± 0.28 mg CE/g, respectively. Both values increased significantly as fermentation progressed, reaching 668.10 ± 8.33 mg GAE/g and 56.99 ± 2.22 mg CE/g, respectively, on day 7 (Figure 1A,B). Kwon et al. [25] reported that TPC levels either increased or remained stable during fermentation in wine prepared from persimmons and inoculated with mixed yeast strains, which was attributed to the extraction of phenolic compounds, such as tannins, from the fruit during alcoholic fermentation. Similarly, Joo et al. [42] observed an increase in gallic acid during fermentation of wine made from Dae-bong persimmons, suggesting that microbial esterases and fermentation-derived organic acids contributed to the release of phenolics. In the present study, the increase in antioxidant compounds was likely due to the enhanced extraction of inherent compounds from persimmon juice and/or the action of enzymes generated during fermentation.

Antioxidant activity was measured using DPPH and ABTS radical scavenging and FRAP reducing power assays (Figure 1C–E). The DPPH and ABTS radical scavenging activities were reported as IC_50_ values, indicating the sample concentration (mg/g) that is necessary to scavenge 50% of free radicals. A lower IC_50_ corresponds to greater antioxidant activity [43]. The DPPH radical scavenging assay showed the highest IC_50_ value of 280.89 ± 7.03 mg/g on day 0, which decreased significantly to 95.79 ± 0.14 mg/g on day 7, indicating enhanced antioxidant activity during fermentation. A comparable pattern was found in the ABTS radical scavenging assay, with the IC_50_ value decreasing from 186.41 ± 3.17 mg/g on day 0 to 97.06 ± 6.40 mg/g on day 7. Trolox, used as a positive control, exhibited IC_50_ values of 41.79 μg/mL (DPPH) and 54.50 μg/mL (ABTS), whereas the persimmon wine samples demonstrated stronger antioxidant activity than Trolox in both assays. Yoon et al. [44] reported that blueberry wine exhibited nearly double the DPPH radical scavenging activity compared to its juice. In the present study, the IC_50_ value on day 0 was nearly three times higher than that on day 7, confirming a marked improvement in antioxidant capacity following fermentation.

Unlike the DPPH and ABTS methods, the FRAP assay measures antioxidant potential by monitoring the conversion of Fe^3+^-TPTZ to Fe^2+^-TPTZ under acidic conditions, a process driven by antioxidants serving as reductants. In this method, higher antioxidant activity results in higher absorbance. The FRAP activity of persimmon wine increased significantly during fermentation, from 0.02 ± 0.00 g FeSO_4_/g on day 0 to 2.54 ± 0.06 g FeSO_4_/g on day 7 (*p* < 0.05). Statistical analysis revealed significant differences among fermentation days, indicating enhanced reducing power over time. This increase is attributed to phenolic compounds with hydroxyl groups, which reduce Fe^3+^ to Fe^2+^ and contribute to radical scavenging activity. The elevated FRAP activity is therefore likely influenced by the increase in TPC and TFC during fermentation [45]. Similarly, Li et al. [46] reported high correlation coefficients (0.939–0.977) between TPC, TFC, and antioxidant activities. In this study, enhanced antioxidant activity appears to be associated with the increase in phenolic and flavonoid compounds over the fermentation period. Zoe et al. [47] reported that the DPPH and ABTS activities increased during alcoholic fermentation, resulting in higher antioxidant activity in persimmon wine compared to puree. Similarly, Ubeda et al. [48] found that the DPPH activity of persimmon wine ranged from 1421 ± 134 to 1870 ± 162 μmol TE/kg, which was approximately 1.2 times higher than that of persimmon juice (1289 ± 22 to 1540 ± 39 μmol TE/kg). These results suggest that as fermentation progresses, both the antioxidant compound content and activity increase significantly, indicating the potential of persimmon wine as a functional alcoholic beverage with health-promoting properties.

## 4. Conclusions

In this study, persimmon wine was produced via inoculation with *Saccharomyces cerevisiae*. Thereafter, its physicochemical properties, organic acid, free sugar, ethanol, methanol, free amino acid, and antioxidant activity were monitored during a 7-day fermentation period. As fermentation progressed, the levels of soluble solids, pH, solids, specific gravity, free sugar, and free amino acids decreased significantly, whereas organic acid, ethanol content, TPC, TFC, and antioxidant activity increased. Notably, methanol content remained below the safety threshold, confirming the safety of the product. This study simultaneously evaluated methanol and antioxidant activity, providing a comprehensive assessment of the safety and functional quality of persimmon wine. The findings suggest that persimmon wine can be utilized as a safe alcoholic beverage with health-promoting functionality. However, this study assessed functionality only through total phenolic and total flavonoid contents and did not perform quantitative analysis of individual phenolic compounds, which represents a limitation. Therefore, future studies should identify and quantify individual phenolic compounds using HPLC to better elucidate the antioxidant characteristics of persimmon wine.

## Figures and Tables

**Table 1:** Changes in physicochemical properties and yeast cell count of persimmon wine during fermentation.

	Fermentation Time (Day)							
	0	1	2	3	4	5	6	7
°Brix (%)	16.27 ± 0.06 ^(1)a(2)^	14.90 ± 0.00 ^b^	8.80 ± 0.00 ^c^	6.77 ± 0.06 ^d^	6.10 ± 0.00 ^e^	5.73 ± 0.06 ^f^	5.70 ± 0.00 ^f^	5.57 ± 0.06 ^g^
pH	5.93 ± 0.01 ^a^	5.70 ± 0.04 ^b^	4.98 ± 0.01 ^c^	4.94 ± 0.01 ^d^	4.86 ± 0.01 ^e^	4.92 ± 0.01 ^ef^	4.91 ± 0.00 ^f^	4.90 ± 0.00 ^g^
Total acidity (%)	0.12 ± 0.01 ^c^	0.11 ± 0.00 ^c^	0.40 ± 0.01 ^a^	0.36 ± 0.01 ^b^	0.41 ± 0.01 ^a^	0.35 ± 0.01 ^b^	0.35 ± 0.03 ^b^	0.41 ± 0.03 ^a^
Yeast cell counts (log CFU/mL)	6.63 ± 0.22 ^e^	7.43 ± 0.18 ^c^	8.14 ± 0.06 ^a^	7.75 ± 0.07 ^b^	7.39 ± 0.12 ^c^	7.13 ± 0.04 ^d^	7.13 ± 0.05 ^d^	7.12 ± 0.03 ^d^
Solid (%)	16.68 ± 0.08 ^a^	13.56 ± 0.01 ^b^	5.66 ± 0.03 ^c^	3.13 ± 0.06 ^d^	2.24 ± 0.02 ^e^	1.84 ± 0.03 ^g^	1.91 ± 0.01 ^g^	2.05 ± 0.09 ^f^
Specific gravity	1.118 ± 0.019 ^a^	1.053 ± 0.003 ^b^	1.018 ± 0.014 ^c^	0.988 ± 0.013 ^c^	0.997 ± 0.018 ^c^	1.008 ± 0.015 ^c^	0.992 ± 0.054 ^c^	1.005 ± 0.029 ^c^

^(1)^ Results are expressed as mean ± SD (*n* = 3). ^(2)^ Different letters indicate significant differences according to Duncan’s test (*p* < 0.05).

**Table 2:** Changes in organic acids, free sugars, ethanol, and methanol contents in persimmon wine during fermentation.

		Fermentation Time (Day)							
Ingredient ^(1)^		0	1	2	3	4	5	6	7
Organic acid (mg/mL)	OA	0.03 ± 0.00 ^(2)^	nd	nd	nd	nd	nd	nd	nd
	CA	0.59 ± 0.00	0.54 ± 0.02	nd	nd	nd	nd	nd	nd
	SA	1.05 ± 0.05 ^c(3)^	1.13 ± 0.03 ^bc^	0.99 ± 0.01 ^c^	1.55 ± 0.45 ^a^	1.28 ± 0.00 ^abc^	1.49 ± 0.21 ^a^	1.43 ± 0.01 ^ab^	1.29 ± 0.05 ^abc^
	LA	nd ^(4)^	1.07 ± 0.01 ^c^	2.40 ± 0.01 ^b^	2.70 ± 0.23 ^a^	2.60 ± 0.01 ^a^	2.61 ± 0.00 ^a^	2.60 ± 0.00 ^a^	2.75 ± 0.01 ^a^
	FA	0.12 ± 0.00 ^a^	0.12 ± 0.00 ^a^	0.08 ± 0.00 ^b^	0.08 ± 0.00 ^c^	0.07 ± 0.00 ^d^	0.07 ± 0.00 ^e^	0.06 ± 0.00 ^fg^	0.06 ± 0.00 ^g^
	AcOH	nd	0.53 ± 0.00 ^c^	1.05 ± 0.17 ^b^	1.24 ± 0.19 ^b^	1.17 ± 0.18 ^b^	1.09 ± 0.01 ^b^	1.10 ± 0.01 ^b^	1.58 ± 0.06 ^a^
	Total	1.76 ± 0.05 ^a^	3.39 ± 0.05 ^b^	4.52 ± 0.18 ^c^	5.57 ± 0.77 ^d^	5.12 ± 0.19 ^d^	5.26 ± 0.21 ^d^	5.19 ± 0.02 ^d^	5.67 ± 0.06 ^d^
Free sugar (mg/mL)	Glu	66.06 ± 0.47 ^a^	58.93 ± 0.08 ^b^	15.64 ± 0.01 ^c^	6.24 ± 0.01 ^d^	nd	nd	nd	nd
	Fru	84.27 ± 0.36 ^a^	80.37 ± 0.05 ^b^	32.14 ± 0.06 ^c^	11.67 ± 0.00 ^d^	2.37 ± 0.10 ^e^	1.16 ± 0.01 ^f^	nd	nd
	Total	150.33 ± 0.84 ^a^	139.24 ± 0.13 ^b^	47.78 ± 0.05 ^c^	17.91 ± 0.01 ^d^	2.37 ± 0.10 ^e^	1.16 ± 0.01 ^f^	nd	nd
Ethanol (%, *v*/*v*)		nd	0.65 ± 0.01 ^g^	5.81 ± 0.00 ^f^	7.20 ± 0.03 ^e^	8.16 ± 0.03 ^c^	8.26 ± 0.04 ^b^	8.35 ± 0.03 ^a^	7.93 ± 0.00 ^d^
Methanol (%, *v*/*v*)		0.046 ± 0.00 ^c^	0.039 ± 0.00 ^e^	0.049 ± ^d^	0.049 ± 0.00 ^bc^	0.042 ± 0.00 ^d^	0.051 ± 0.00 ^a^	0.051 ± 0.00 ^bc^	0.050 ± 0.00 ^bc^

^(1)^ Abbreviations: OA, oxalic acid; CA, citric acid; SA, succinic acid; LA, lactic acid; FA, fumaric acid; AcOH, acetic acid; Glc, glucose; Fru, fructose. ^(2)^ Results are expressed as means ± SD (*n* = 3). ^(3)^ Different letters indicate significant differences according to Duncan’s test (*p* < 0.05). ^(4)^ Values below the detection limit are expressed as nd (not detected).

**Table 3:** Changes in free amino acid contents in persimmon wine during fermentation.

		Fermentation Time (Day)							
Ingredient ^(1)^		0	1	2	3	4	5	6	7
Free amino acid (μg/mL)	His	33.82 ± 4.71 ^(2)^	12.85 ± 18.17	nd	nd	nd	nd	nd	nd
	Arg	142.34 ± 38.65	68.07 ± 2.88	nd	nd	nd	nd	nd	nd
	Thr	94.71 ± 30.82	60.40 ± 1.39	nd	nd	nd	nd	nd	nd
	Pro	254.76 ± 92.39 ^a(3)^	171.72 ± 84.23 ^b^	63.92 ± 32.97 ^b^	92.41 ± 1.68 ^b^	86.37 ± 17.23 ^b^	74.24 ± 12.62 ^c^	46.85 ± 19.90 ^c^	62.97 ± 46.85 ^c^
	Ile	13.21 ± 7.97	3.33 ± 4.71	nd	nd	nd	nd	nd	nd
	Leu	13.67 ± 3.83	nd ^(4)^	nd	nd	nd	nd	nd	nd
	Phe	10.93 ± 4.16	10.04 ± 0.58	nd	nd	nd	nd	nd	nd
	Total	563.43 ± 182.53 ^a^	326.40 ± 60.44 ^b^	63.92 ± 32.97 ^c^	92.41 ± 1.68 ^c^	86.37 ± 17.23 ^c^	74.24 ± 12.62 ^c^	46.85 ± 19.90 ^c^	62.97 ± 46.85 ^c^

^(1)^ Abbreviations: His, histidine; Arg, arginine; Thr, threonine; Pro, proline; Ile, isoleucine; Leu, leucine; Phe, phenylalanine. ^(2)^ Results are expressed as means ± SD (n = 3). ^(3)^ Different letters indicate significant differences according to Duncan’s test (*p* < 0.05). ^(4)^ nd indicates that the compound was not detected.

## Data Availability

The original contributions presented in this study are included in the article. Further inquiries can be directed to the corresponding author.
